# Cause of Death in Intestinal Obstruction

**Published:** 1916-07

**Authors:** 


**Cause of Death in Intestinal Obstruction.** Frederick Hagler and Major G. Seelig in a review of 39 articles from periodic literature, Interstate Med. Jour. conclude that the inanition theory is not correct, death occurring too soon, although it must be admitted that death occurs earlier from high than from low obstruction. In certain cases, with much vomiting, death may be due to dehydration. The nervous theory is plausible but the tachycardia, low blood pressure, dilatation of splanchnic vessels and collapse do not differ materially from the phenomena of approaching death in various other conditions, including toxaemias in general. Bacterial infection is denied, a priori, by the more rapid death in high than in low obstruction and by the fact that experimental exclusion of an intestinal loop is not incompatible with indefinite prolongation of life. Intoxication, by bacterial and by metabolism processes is discussed at great length. There is much difference of opinion and some apparent contradictions of evidence. Probably death is due to no one cause.

